# Patient-reported Outcome Measures of Revision Total Hip Arthroplasty for Prosthetic Joint Infection is not Inferior to Aseptic Revision Total Hip Arthroplasty

**DOI:** 10.5704/MOJ.2011.012

**Published:** 2020-11

**Authors:** JBT Lim, HN Pang, KJD Tay, SL Chia, SJ Yeo, NN Lo

**Affiliations:** Department of Orthopaedic Surgery, Singapore General Hospital, Singapore

**Keywords:** revision total hip arthroplasty, prosthetic joint infection, aseptic revision total hip arthroplasty, outcomes, satisfaction

## Abstract

**Introduction:**

This study aims to investigate whether patients undergoing two-stage revision total hip arthroplasty (THA) for prosthetic joint infection (PJI) and one-stage revision THA for aseptic reasons have similar clinical outcomes and patient satisfaction during their post-operative follow-up. We hypothesise that the two-stage revision THA for PJI is associated with poorer outcomes as compared to aseptic revision THA.

**Materials and Methods:**

We reviewed prospectively collected data in our tertiary hospital arthroplasty registry and identified patients who underwent revision THA between 2001 and 2014, with a minimum of two years follow-up. The study group (two-stage revision THA for PJI) consists of 23 patients and the control group (one-stage revision THA for aseptic reasons) consists of 231 patients. Patient demographics, Western Ontario and McMaster Universities Arthritis Index (WOMAC), Oxford Hip Score (OHS), Short Form-36 (SF-36) scores and patient reported satisfaction were evaluated. Student’s t-test was used to compare continuous variables between the two groups. Statistical significance was defined as p <0.05.

**Results:**

The pre-operative demographics and clinical scores were relatively similar between the two groups of patients. At two years, patients who underwent revision THA for PJI reported a better WOMAC Pain Score and OHS as compared to aseptic revision THA. A similar proportion of patients were satisfied with their results of surgery in both groups (p=0.093).

**Conclusions:**

Although patients who underwent revision THA for PJI had poorer pre-operative functional scores (WOMAC function and SF-36 PF), at two years follow-up, these two groups of patients have comparable post-operative outcomes. Interestingly, patients who had revision THA for PJI reported a better clinical outcome in terms of OHS and WOMAC Pain score as compared to the aseptic group. We conclude that the revision THA for PJI is not inferior to aseptic revision THA in terms of patient satisfaction and clinical outcomes.

## Introduction

Primary total hip arthroplasty (THA) is a frequently performed orthopaedic surgery that significantly improves a patient’s quality of life and achieves high patient satisfaction rates^1-3^. The long-term outcomes of THA are favourable, with approximately 90% of patients reporting that their expectations have been met by surgery and that they are satisfied with the results of their surgery^3^. As the number of patients with medically diagnosed arthritis increases, so does the number of primary THA. Similarly, the rates of revision THA have risen^4^. It has been reported in the literature that approximately 10 to 18% of all THA interventions are revision procedures for aseptic reasons such as aseptic loosening, wear, instability with recurrent dislocations, periprosthetic fractures as well as septic THA revision^4^.

Revision THA as compared to primary THA is associated with significantly higher complications and mortality rates, less improvement in functional outcome and quality of life, as well as lower patient satisfaction^5-7^. It has been postulated that technical factors, poorer quality of bone and patient-related factors such as older age, more comorbid conditions and poorer pre-operative baseline pain scores are confounding factors for less favourable results for revision THA as compared to primary THA^7-9^. Common reasons for revision THA in the United States is aseptic mechanical loosening, instability/dislocation, followed by periprosthetic joint infection (PJI)^10,11^. For septic revision THA, a two-stage revision is the most widely accepted and performed intervention, with a high infection eradication rate that exceeds 90% in most studies^12-16^. This two-stage procedure involves a first stage procedure of implant replacement with an antibiotic-loaded cement spacer, followed by a second stage procedure of re-implantation with a cemented or uncemented prosthesis.

Despite the ability to control deep-seated infection in patients with PJI with two-stage revision THA, these patients often report poorer functional outcomes following revision surgery as compared to those who have undergone aseptic revision THA^16^. There have been studies in the literature^17,18^ comparing outcome measures between revision total knee arthroplasty for PJI and aseptic reasons. However, there is little literature available that compares patient-reported outcome measures and patient satisfaction between the two-stage revision THA for PJI and the one-stage revision THA for aseptic reasons such as instability and loosening^16^.

This study aims to compare the patient-reported outcome measures for patients undergoing revision THA for PJI and aseptic revision THA, in order to evaluate if the two groups of patients have similar clinical outcomes and patient satisfaction in their two years follow-up post-operatively. The hypothesis of this study is that the two-stage revision THA for PJI is associated with poorer outcomes as compared to aseptic revision THA.

## Materials and Methods

Centralised institutional review board has approved this study (CIRB: 2016/2422). This is retrospective study of prospectively collected data of a tertiary hospital arthroplasty registry. Seven adult reconstruction trained consultant orthopaedic surgeons within the hospital performed all of the revision THA operations for this study. During the 13 years study period between year 2001 and 2014, we identified patients who underwent revision THA with a minimum of 2 years follow-up. A total of 254 patients were identified for the study. The study group (two-stage revision THA for PJI) consists of 23 patients and the control group (one-stage revision THA for aseptic reasons) consists of 231 patients. Power analysis was performed to ensure that our study sample size is adequate. A total sample size required was 147 with an effect size of 0.3 and achieving a 95% power with an alpha risk of 5%. A larger sample for the aseptic revision THA was chosen to increase the power of the study.

The two-staged revision THA for PJI will typically involve explanting the total hip implant with insertion of an articulating antibiotic cement spacer during the Stage 1 procedure. During the Stage 2 procedure, the antibiotic cement spacer will be removed and implantation of total hip implants for both the acetabular and femoral components will be performed.

We evaluated patient demographics such as age, gender and body mass index (BMI), Charlson age-comorbidity index from the clinical records as well as database of the arthroplasty registry. Pre-operative baseline scores and postoperative scores were obtained at six-months and two years follow-up. For the PJI group, the pre-operative scores were obtained prior to the first-stage revision THA. Patient-reported outcome measures were assessed using a self-administered patient questionnaire that is given with a validated scoring system: Western Ontario and McMaster Universities Arthritis Index (WOMAC), Oxford Hip Score (OHS) and Short Form-36 (SF-36) Health Survey. The OHS is based on the 12 items on daily activities, and each item is scored from 1 - 5, with 1 representing the best outcome/least symptoms and 5 representing the worst outcome/most symptoms. The WOMAC scoring is based on higher scores indicating that the patient reports less pain, less stiffness and less functional limitations. Post-operatively at six months and two years, we also evaluated patients’ opinions as to whether the surgery met their expectations and whether they were satisfied with the results of their surgery using two questions adopted from the validated North American Spine Society (NASS) Questionnaire, which were (1) “Has the surgery met your expectations so far?” and (2) “How would you rate the overall results of surgery?”. For Question 1, patients had the choice of selecting from the following answers: (1) Yes, totally; (2) Yes, almost totally; (3) Yes, quite a bit; or (4) More or less; (5) No, not quite; (6) No, far from it or (7) No, not at all. For Question 2, patients had the choice of selecting from the following answers: (1) Excellent; (2) Very good; or (3) Good; (4) Fair; (5) Poor or (6) Terrible. Patients were defined as either having had their expectations met by their surgeries (responses to Question 1 = 1-4) or not having had their expectations met by their surgeries (responses to Question 1 = 5-7). Patients were defined as either satisfied (responses to Question 2 = 1-3) or dissatisfied (responses to Question 2 = 4-6). The mean follow-up range is 8.0 ± 3.7 (range 2-16) years for those with PJI two-staged revisions. The mean follow-up range is 6.0 ± 1.6 (range 2-13.2) years for those with aseptic revision THA. The continuous variables of our study were presented as mean ± standard deviation. Comparisons were made by use of unpaired t-test between the aseptic revision THA and revision THA for PJI groups. ANOVA was used to make comparisons between the pre-operative and post-operative follow-up clinical scores at six months and two years among the group. Z-score was used to compare categorical variables between the two groups, such as the gender and proportion of patients satisfied with their results of their surgery.

For all analyses, statistical significance was defined as p-value of 0.05 or less. All statistical analysis was performed using SPSS version 23 [SPSS, Inc., Chicago, Illinois].

## Results

The pre-operative demographics of our patients are shown in (Table I). Both the control and study group patients had similar age at time of surgery, Charlson comorbidity index, BMI pre-operatively. The study group of revision THA for PJI had a mean age of 59.6, while the aseptic revision THA group had a mean age of 64.1 years old (p=0.117). The BMI of the study group was 26.4 versus 26.5 for the control group (p=0.929). The mean Charlson comorbidity index for both the groups of patients was 2.9 (p=0.971). From our study, the main reasons for aseptic revision THA include aseptic loosening (183 patients), instability/dislocation (34 patients), component failure (7 patients) and periprosthetic fracture of THA (7 patients). All of the patients had deep-seated periprosthetic joint infection. The type of infective organisms involved for the PJI group includes: nine patients with methicillin-sensitive staphylococcus aureus (MSSA), seven patients with methicillin-resistant staphylococcus aureus (MRSA), four patients with enterococcus infection, one patient with alpha-hemolytic streptococcus, one patient with group B streptococcus (GBS) and one patient with tuberculosis infection. The mean timeline from first-staged revision to second-staged revision for PJI patients is 5.3 (± 3.3) months. There were no septic recurrences for the PJI patients within the two years of follow-up. Articulating spacer was used for all of the first-staged revision THA. After second-staged revision THA for the PJI group, two patients had recurrent dislocations and subsequently one of these two patients had a constrained liner exchange at 1 week post-operatively and another patient had recurrent dislocation with change of liner and femoral head at two years post-operatively (Table II). The two patients were not excluded from the study. The complications for the aseptic revision are listed in (Table II) with 4 intra-operative complications, 19 early and 13 late complications. The number of complications were not significant between both groups of patients (p=0.379).

**Table I:** Demographics of patients who underwent revision total hip arthroplasty (* = significance at p < 0.05)

Pre-operative clinical details	Aseptic revision THA	Revision THA due to periprosthetic joint infection	P-values
Number of Patients	231	23	N.A.
Gender	Male patients = 83	Male patients = 10	0.472
	Female patients = 148	Female patients = 13	
Age of surgery	64.1 ± 12.6	59.6 ± 11.3	0.117
Body Mass Index (BMI)	26.4 ± 4.9	26.5 ± 5.4	0.929
Mean Charlson Age	2.9 ± 1.6	2.9 ± 1.8	0.971
Co-morbidity Index			
Mean number of years follow-up (Years)	6.0 ± 1.6 (Range: 2 – 13.2)	8.0 ± 3.7 (Range: 2 – 16)	N.A.
Reasons for Revision THA (n= number of patients)	Wear and aseptic Loosening (n=183) (72%)	Prosthetic joint infection (n=23) (9%)	N.A.
	Instability / Dislocation (n=34) (13.4%)		
	Component Failure (n=7) (2.8%)		
	Periprosthetic Fracture (n=7) (2.8%)		
Surgical approach for revision THA	Posterior approach (n=156)	Posterior approach (n=16)	N.A.
	Hardinge approach (n= 75)	Hardinge approach (n=7)	

**Table II:** Complications requiring secondary surgical procedure after index revision total hip arthroplasty (* = significance at p < 0.05)

Post-operative complications	Aseptic revision THA periprosthetic joint infection	Revision THA due to	P-values
Number of patients with post-operative complications	(Early < 2 years ; Late > 2 years)	4 intra-operative, 19 Early and 13 late	1 early and 1 late 0.379
Type of Intra-operative complications	Intra-op arterial bleeding managed with angiogram and embolisation 3 Intra-operative periprosthetic fracture	Nil	
Type of Early complications	5 patients with periprosthetic fracture (1 at 1 week 1 at 2 weeks and 3 at 1 month) 5 patients with instability managed with revision (1 week, 2 at 1 month, 1 at 2 months, 1 at 3 months) 4 patients with infection managed with debridement and arthrotomy (2 at 1 week 1 at 3 months, 1 at 6 months) 2 patients with seroma managed with debridement (2 at 9 months) 2 patients with early failure with loosening of stem managed with revision (1 patient at 3 months 1 at 1 year) 1 patient with deep PJI at 1 year managed with 2 staged revision	1 patient with recurrent dislocation at 1 week postoperative managed with change of liner	
Type of Late complications	5 patients with periprosthetic fracture (1 at 2 years, 2 at 5 years, 1 at 8 years and 1 at 15 years) 3 patients with Deep infection managed with 2 staged revision (1 at 2 years, 1 at 5 years, 1 at 14 years) 7 patients with aseptic loosening (1 at 2 years, 1 at 2.2 years, 2 at 6 years, 1 at 6.3 years, 1 at 11 years, 1 at 13 years)	1 patient with recurrent dislocation at 2 years managed with change of liner and femoral head	

The pre-operative clinical scores for both groups of patients were compared (Table III). The pre-operative clinical scores of patients are relatively similar between the two groups of patients. The study group and control group had similar WOMAC Scores (pain and stiffness), Oxford Hip scores and SF-36 (physical role functioning, bodily pain, general health, vitality, social functioning, emotional role functioning, mental health, physical component score and mental component score) (p>0.05). The aseptic revision THA patients had significantly better baseline functional scores (WOMAC function and SF-36 sub-domain of physical functioning (PF)) than the patients who underwent revision THA for PJI. The WOMAC function score for the aseptic revision THA group was 46.2 versus 30.3 for the group of revision THA for PJI (p=0.010). The SF-36 (PF) for the aseptic revision THA group was also significantly better (27.5 versus 14.5; p=0.024).

**Table III:** Comparison of pre-operative clinical scores for both groups of patients who underwent revision total hip arthroplasty (* = significance at p < 0.05)

Pre-operative clinical scores	Aseptic revision THA	Revision THA due to periprosthetic joint infection	P-values
WOMAC Score (Pain)	57.1 ± 30.2	54.3 ± 41.2	0.763
WOMAC Score (Stiffness)	73.6 ± 30.9	76.2 ± 36.4	0.717
WOMAC Score (Function)	46.2 ± 26.9	30.3 ± 27.2	0.010*
Oxford Hip Score	38.6 ± 11.7	42.2 ± 12.8	0.179
SF-36 (Physical Functioning)	27.5 ± 25.4	14.5 ± 22.2	0.024*
SF-36 (Physical Role Functioning)	19.9 ± 36.4	8.3 ± 26.6	0.076
SF-36 (Bodily Pain)	33.8 ± 26.7	29.1 ± 24.8	0.442
SF-36 (General Health)	66.4 ± 21.8	69.1 ± 19.8	0.592
SF-36 (Vitality)	63.1 ± 23.7	66.7 ± 20.7	0.509
SF-36 (Social Functioning)	42.6 ± 37.9	27.3 ± 40.1	0.080
SF-36 (Emotional Role Functioning)	75.3 ± 41.4	57.1 ± 50.7	0.126
SF-36 (Mental Health)	72.0 ± 21.7	65.1 ± 20.8	0.163
SF-36 Physical component Score (PCS)	36.9 ± 21.4	30.2 ± 16.9	0.163
SF-36 Mental component Score (MCS)	63.3 ± 21.7	54.1 ± 23.5	0.068

The six months follow-up scores were compared between both groups of patients (Table IV). At six months, there was no significant difference between the WOMAC Function score between the study and control group (67.6 versus 72.3; p=0.369). The study group patients had poorer SF-36 PF as compared to the control group (25.0 versus 48.7; p=0.037). This trend was also seen pre-operatively. The SF-36 social role functioning for the study group was also poorer than the control group (48.4 versus 70.9; p=0.019). In addition, the SF-36 physical component score (PCS) for the study group was also poorer than the control group (44.6 versus 57.3; p=0.047). The other clinical scores were similar for the two groups with p>0.05.

**Table IV:** Six months follow-up scores with comparison of clinical outcomes between the two groups of patients who underwent revision total hip arthroplasty (* = significance at p < 0.05)

6 Months Clinical Score	Aseptic revision THA	Revision THA due to periprosthetic joint infection	P-values
WOMAC Score (Pain)	90.1 ± 17.0	92.9 ± 11.3	0.524
WOMAC (Stiffness)	87.6 ± 20.2	84.1 ± 18.3	0.500
WOMAC (Function)	72.3 ± 20.2	67.6 ± 20.9	0.369
Oxford Hip Score	23.9 ± 9.9	26.0 ± 9.3	0.423
SF-36 (Physical Functioning)	49.0 ± 26.5	35.9 ± 30.0	0.064
SF-36 (Physical Role Functioning)	48.7 ± 46.3	25.0 ± 39.8	0.037*
SF-36 (Bodily Pain)	62.9 ± 27.8	53.5 ± 26.9	0.193
SF-36 (General Health)	68.4 ± 21.1	64.0 ± 21.2	0.424
SF-36 (Vitality)	69.1 ± 20.4	71.9 ± 16.8	0.601
SF-36 (Social Functioning)	70.9 ± 35.5	48.4 ± 44.2	0.019*
SF-36 (Emotional Role Functioning)	88.4 ± 31.1	81.3 ± 40.3	0.396
SF-36 (Mental Health)	79.0 ± 16.7	72.3 ± 15.9	0.121
SF-36 Physical component Score (PCS)	57.3 ± 24.4	44.6 ± 23.5	0.047*
SF-36 Mental component Score (MCS)	76.9 ± 19.2	68.6 ± 22.5	0.102

Table V shows the comparison between the two groups of patients at two years follow-up. At two-years follow-up, patients who underwent revision THA for PJI had similar functional scores (WOMAC function and SF-36 PF) when compared to the aseptic revision THA group. The functional scores for the study group had “caught up” with the control group, despite starting off with a lower pre-operative functional score. At two years follow-up, the WOMAC function score for the study and control groups was 81.7 and 76.2, respectively (p=0.330). The SF-36 PF for the study and control groups was 58.1 and 51.4, respectively (p=0.424). As compared to aseptic revision THA, patients who underwent revision THA for PJI also reported a better WOMAC pain score (99.7 versus 91.3; p<0.001) and OHS (17.5 versus 22.1, p<0.030) (Table V). The patient-reported satisfaction and expectations been met are shown in (Table V). A similar proportion of patients in both groups were satisfied with their results of surgery (p=0.093). However, there were a significantly larger proportion of patients in the aseptic revision THA group who had their expectations met by surgery as compared to those who underwent revision THA for PJI (p=0.005).

**Table V:** Two years follow-up scores with comparison of clinical outcomes between the two groups of patients who underwent revision total hip arthroplasty (* = significance at p < 0.05)

2 Years Follow-up	Aseptic revision THA	Revision THA due to periprosthetic joint infection	P-values
WOMAC Score (Pain)	91.3 ± 17.8	99.7 ± 1.1	<0.001*
WOMAC (Stiffness)	91.3 ± 16.4	95.0 ± 10.8	0.425
WOMAC (Function)	76.2 ± 19.4	81.7 ± 18.1	0.330
Oxford Hip Score	22.1 ± 9.8	17.5 ± 6.4	0.030*
SF-36 (Physical Functioning)	51.4 ± 28.9	58.1 ± 28.8	0.424
SF-36 (Physical Role Functioning)	53.8 ± 45.9	71.2 ± 41.9	0.176
SF-36 (Bodily Pain)	64.7 ± 29.9	75.2 ± 30.6	0.226
SF-36 (General Health)	65.6 ± 22.8	57.9 ± 31.3	0.259
SF-36 (Vitality)	70.1 ± 21.1	73.1 ± 29.1	0.635
SF-36 (Social Functioning)	75.5 ± 34.7	74.1 ± 34.7	0.888
SF-36 (Emotional Role Functioning)	90.2 ± 28.6	92.3 ± 27.7	0.796
SF-36 (Mental Health)	80.0 ± 18.5	80.1 ± 18.5	0.911
SF-36 Physical component Score (PCS)	58.9 ± 26.2	65.6 ± 28.0	0.379
SF-36 Mental component Score (MCS)	78.9 ± 19.4	80.2 ± 25.1	0.838
Percentage of patients whose expectations were met by surgery	81.4%	57.1%	0.005*
Percentage of patients who were satisfied with overall results of surgery	83.5%	71.4%	0.093

The degrees of improvement for both groups of patients were compared at post-operative six months (Table VI) and two years (Table VII) follow-up. At six months, both groups of patients made the same degree of improvement as compared to pre-operatively. However, at two years follow-up, the revision THA group for PJI made a greater degree of improvement as compared to the aseptic revision THA group for WOMAC Pain Score (51.5 versus 33.1, p=0.024), WOMAC Function Score (56.1 versus 28.8, p<0.001), Oxford Hip Score (27.8 versus 16.6, p<0.001), SF-36 physical functioning (48.8 versus 23.6, p=0.032), SF-36 physical role functioning (71.1 versus 34.3, p=0.004), SF-36 bodily pain (54.3 versus 30.3, p=0.033), SF-36 social functioning (60.7 versus 31.9, p=0.002), SF-36 PCS (41.8 versus 22.3, p=0.028) and SF-36 MCS (30.6 versus 16.3, p=0.011).

**Table VI:** Degree of change between pre-operative and six months clinical outcomes between the two groups of patients who underwent revision total hip arthroplasty (* = significance at p < 0.05)

Degree of change for Clinical Score between pre-operative and 6 months score	Aseptic revision THA	Revision THA due to periprosthetic joint infection	P-values
WOMAC Score (Pain)	31.8 ± 32.7	38.4 ± 45.6	0.245
WOMAC Score (Stiffness)	15.2 ± 32.3	8.4 ± 34.1	0.795
WOMAC Score (Function)	24.1 ± 26.3	37.5 ± 31.9	0.052
Oxford Hip Score	14.2 ± 12.6	17.1 ± 15.1	0.313
SF-36 (Physical Functioning)	20.1 ± 25.8	22.8 ± 37.4	0.751
SF-36 (Physical Role Functioning)	27.9 ± 52.1	14.1 ± 55.5	0.213
SF-36 (Bodily Pain)	28.0 ± 32.9	26.3 ± 42.0	0.663
SF-36 (General Health)	3.5 ± 23.7	1.1 ± 27.9	0.471
SF-36 (Vitality)	7.6 ± 27.2	5.6 ± 20.9	0.896
SF-36 (Social Functioning)	27.5 ± 40.2	25.1 ± 53.8	0.878
SF-36 (Emotional Role Functioning)	13.8 ± 50.3	18.8 ± 54.4	0.725
SF-36 (Mental Health)	7.3 ± 23.4	7.8 ± 20.3	0.734
SF-36 Physical component Score (PCS)	19.9 ± 25.1	15.5 ± 31.9	0.848
SF-36 Mental component Score (MCS)	14.1 ± 23.8	14.3 ± 27.6	0.189

**Table VII:** Degree of change between pre-operative and two years clinical outcomes between the two groups of patients who underwent revision total hip arthroplasty (* = significance at p < 0.05)

Degree of change for Clinical Score between pre-operative and 2 years scores	Aseptic revision THA THA	Revision THA due to periprosthetic joint infection	P-values
WOMAC Score (Pain)	33.1 ± 32.8	51.5 ± 43.9	0.024*
WOMAC Score (Stiffness)	18.5 ± 32.3	16.9 ± 32.6	0.614
WOMAC Score (Function)	28.8 ± 27.7	56.1 ± 25.6	<0.001*
Oxford Hip Score	16.6 ± 12.3	27.8 ± 10.9	<0.001*
SF-36 (Physical Functioning)	23.6 ± 29.4	48.8 ± 36.9	0.032*
SF-36 (Physical Role Functioning)	34.4 ± 51.9	71.1 ± 41.9	0.004*
SF-36 (Bodily Pain)	30.3 ± 34.9	54.3 ± 34.7	0.033*
SF-36 (General Health)	0.8 ± 25.8	7.3 ± 33.9	0.476
SF-36 (Vitality)	7.7 ± 24.2	5.8 ± 33.1	0.784
SF-36 (Social Functioning)	31.9 ± 46.1	60.7 ± 38.5	0.002*
SF-36 (Emotional Role Functioning)	16.7 ± 49.2	38.5 ± 65.0	0.204
SF-36 (Mental Health)	9.1 ± 22.8	17.2 ± 36.6	0.330
SF-36 Physical component Score (PCS)	22.3 ± 26.9	41.8 ± 29.3	0.028*
SF-36 Mental component Score (MCS)	16.3 ± 23.8	30.6 ± 35.9	0.011*

## Discussion

THA has been an extremely successful operation for symptomatic relief of end-stage hip arthritis, but a proportion of failures are inevitable^19^. There are several modes of failure for the implanted hip leading to revision THA^20-24^. From our study, we report the following causes for revision THA in our patients: 72% were due to wear and aseptic loosening, 13.4% due to instability/dislocation, 2.8% due to component failure. 2.8% due to periprosthetic fracture and 9% due to PJI. From the literature^20-24^, early failures are often directly related to surgical technique, such as component malposition, soft tissue laxity leading to instability of the THA implant and operative field inoculation with bacteria causing deep infection and PJI. For late failures, aseptic loosening continues to represent a significantly predominant mode of failure for primary THA, accounting for approximately 52% of revision cases^20-24^.

The use of a two-stage exchange procedure with antibiotic-loaded cement for chronically infected THA together with the use of adjuvant antibiotic therapy has been successful in eradication of the infection with achievement of satisfactory outcomes^25-29^. The use of a spacer will allow a delay of several months between the two stages with a distinct advantage, since the control of deep-seated infection may sometimes require several months of post-operative antibiotic therapy^27-29^.

Revision THA has its implications of burden of care for the healthcare system. The direct medical costs of revision THA for aseptic loosening was reported to be 4.8 folds higher than that of primary THA^29^. As compared to aseptic revision THA, septic revision THA requires more costly medical treatment such as the need for long term intravenous antibiotic therapy, which has been reported to be approximately 2 to 2.8 times higher than that for aseptic THA^16,30,31^.

There are several papers in the literature^16,17,28,29^ that have compared revision total knee arthroplasty (TKA) for septic versus aseptic reasons with conflicting results. Meek *et al* (2004)^28^ concluded that the two-stage exchange procedure for septic TKA provided comparable patient satisfaction and functional outcome (WOMAC, Oxford-12, SF-12, patient satisfaction data and range of movement). In a similar study by Patil *et al* (2010)^29^, patients who we operated on for septic revision TKA had better outcomes compared to those with aseptic revision TKA, with more improvement in their KSS (p=0.004) and Function Scores (p=0.02). It has been postulated that perhaps it is not that septic revisions are doing better than expected, but that the aseptic revisions may often do worse than it is presently assumed^29^. On the other hand, there have also been studies^17,18^ showing inferior results for septic revision arthroplasty as compared to aseptic revision arthroplasty. However, there is little literature available from prospective comparative studies about how quality of life, patient function and satisfaction rates compare between septic versus aseptic revision THA, which our study aims to contribute to the literature.

In our study, patients who underwent revision THA for PJI had poorer pre-operative functional scores (WOMAC function and SF-36 PF). However, at two years follow-up, the septic revision THA had “caught up” in their functional scores and both groups of patients had comparable postoperative outcomes. Interestingly, our patients who had revision THA for PJI also reported better clinical outcome in terms of OHS and WOMAC Pain score as compared to the aseptic group at two years follow-up. There was also a greater degree of improvement made for WOMAC (Pain, function), OHS, SF-36 (physical functioning, physical role functioning, bodily pain, social functioning, PCS and MCS) for the patients who underwent revision THA for PJI at two years follow-up as compared to pre-operatively. This finding is similar to that of studies, which have shown that septic revision, arthroplasty provides better outcome than aseptic revision arthroplasty^16,28,29^. Aseptic revisions are often associated with severe bone defects from osteolysis or from periprosthetic fractures, making the reconstruction technically challenging. Revision surgeries for aseptic failures are also associated with problems of loss of soft tissue integrity resulting from recurrent dislocations or from wear debris meditated inflammatory osteolysis^32^. However, for revision THA due to PJI, the optimal outcomes are more dependent on the successful eradication of infection. In a two-staged revision THA for PJI, which has high success in bacteria eradication, with no recurrence of deep infection after re-implantation during stage two revision THA and lesser bone defect compared to aseptic revision THA, which could account for the better clinical outcome at two years follow-up.

In terms of patient-reported satisfaction, the patients in our study who underwent revision THA for PJI were equally satisfied compared to the aseptic revision THA patients. The relatively equal rates of patient satisfaction at two years follow-up could be correlated to both patients groups obtaining significant improvement in most aspects of the patient-reported outcome measures with good pain relief and functional outcome. However, a significantly larger proportion of patients did not have their expectations met by surgery in the group of patients who underwent revision THA for PJI as compared to the control group. This could be related to the more extensive, lengthy two-staged surgery required for bacteria eradication prior to re-implantation and also more costly overall medical treatment for two-staged revision THA for PJI. With a longer and more costly treatment for two-staged revision THA for PJI, a larger proportion of patients may not have their expectations met by the surgery, as compared to a one-staged aseptic revision THA. However, the authors acknowledge that the poorer expectation for the PJI group could reflect the weakness of the categorical survey questionnaire, which may not be responsive enough to truly measure the patients’ expectation of their surgery post-operatively for the two groups of non-homogenous patients.

We report several limitations of our study. This is a retrospective study with a smaller number of patients with septic revision THA as compared to the number of patients with aseptic revision THA. The outcome follow-up period of two years is also relatively short and a more prolonged observation is required to provide information on the long-term outcome in these patients. However, it has been reported that the outcomes of revision THA will plateau at two years follow-up, and our study assumed that these patients with revision THA for aseptic and septic reasons had achieved their maximum clinical improvement^33^.

## Conclusion

We conclude that revision THA achieved satisfactory functional outcomes and patient-reported satisfaction. In our study, revision THA for PJI achieved a better clinical outcome in terms of OHS and WOMAC Pain score as compared to aseptic revision THA at two years follow-up. There was also a greater degree of improvement made for WOMAC Score (Pain and function), SF-36 (physical functioning, physical role functioning, bodily pain, social functioning, PCS and MCS) for the patients who underwent revision THA for PJI. In terms of patient-reported satisfaction and clinical outcomes, revision THA for PJI is not inferior to that of aseptic revision THA. A good outcome from revision for aseptic failures requires optimal management of bone loss and restoration of soft tissue balance. However, for revision THA in septic failures, the optimal outcomes are more dependent on the successful eradication of infection. We postulate in our study, that the smaller bone defect and no recurrence of deep infection requiring re-revision THA for the PJI patients, could have accounted for the greater degree of improvement and better clinical outcome as compared to aseptic revision THA at two years follow-up.
